# Characteristics and Practices of High-Performing Centers in Organ Donor Identification and Referral: A Qualitative Study

**DOI:** 10.1177/20543581241276362

**Published:** 2024-09-21

**Authors:** Leahora Rotteau, Samuel Vaillancourt, Mercedes Magaz, Lisha Lo, Brian M. Wong, Jehan Lalani, Sam D. Shemie, Samara Zavalkoff

**Affiliations:** 1 Centre for Quality Improvement and Patient Safety, Temerty Faculty of Medicine, University of Toronto, ON, Canada; 2 Institute for Healthcare Policy, Evaluation and Management, Temerty Faculty of Medicine, University of Toronto, ON, Canada; 3 Department of Emergency Medicine, Li Ka Shing Knowledge Institute, St. Michael’s Hospital, Unity Health Toronto, ON, Canada; 4 Department of Medicine, Temerty Faculty of Medicine, University of Toronto, Toronto, ON, Canada; 5 Division of Critical Care, Department of Pediatrics, McGill University, Montreal, QC, Canada; 6 The Research Institute of the McGill University Health Centre, Montreal, QC, Canada; 7 Organ and Tissue Donation and Transplantation, Canadian Blood Services, Ottawa, ON, Canada

**Keywords:** organ donation, critical care, qualitative research, positive deviance, quality improvement

## Abstract

**Background:**

The identification and referral (ID&R) of potential organ donors to provincial organ donation organizations (ODOs) is a critical first step in the organ donation process. However, even in provinces with mandatory referral legislation, there remains variability in ID&R rates across critical care units, with some units demonstrating high performance despite experiencing similar constraints associated with existing structures, policies, and practices.

**Objective:**

We sought to identify the enablers and specific strategies that high-performing critical care units leveraged to achieve their exceptional performance.

**Design:**

We conducted a descriptive qualitative study to inform ID&R improvement efforts as part of a positive deviance initiative.

**Setting:**

We identified three high-performing critical care units as study sites.

**Participants:**

Clinicians working in identified critical care units.

**Methods:**

At each site, we interviewed clinical team members about their perceptions and experiences of ID&R. Data analysis followed a thematic analysis approach.

**Results:**

We outline three themes describing how the high-performing hospitals achieve strong ID&R practices. First, all units demonstrated a high degree of integration between the concepts of high-quality end-of-life care and organ donation. Team members were consistently notified of successful transplants stemming from their unit, and all missed ID&Rs were tracked and discussed. Second, participants described a team approach with strong medical leadership, where all team members embrace their role in ensuring that no potential donor is missed. Finally, the units adopted strategies to support and simplify ID&R such as collectively simplifying triggers for referral, developing strong working relationships with provincial donor coordinators, and creating informal avenues of communication between clinicians and donor coordinators.

**Limitations:**

The lack of comparable data for potential organ donor referral rates across Canada impacted our ability to identify high-performing hospitals based on data. Instead, we contacted the ODOs directly to identify high-performing units that met our criteria. Second, our study sample was limited to three hospital sites from three different provinces and the three hospitals perform organ recovery and transplant on-site.

**Conclusion:**

Critical care units can adopt strategies and implement interventions to support ID&R improvement efforts. We provide examples informed by this study. We also highlight considerations that require attention when engaging in this work such as ensuring that all team members are aware of changes in care plans and physicians consistently engage in discussions about organ donation. Local medical leadership is critical to supporting these changes.

## Abrege

**Contexte:**

L’identification et l’aiguillage (ID+AIG) des donneurs d’organes potentiels vers les organismes provinciaux de don d’organes (OPDO) constituent une première étape essentielle du processus de don d’organes. Toutefois, même dans les provinces où la loi oblige l’aiguillage des donneurs potentiels, les taux d’ID+AIG varient entre les unités de soins intensifs, certaines affichant un rendement élevé malgré des contraintes similaires associées aux structures, aux politiques et aux pratiques existantes.

**Objectif:**

Nous cherchions à identifier les facilitateurs et les stratégies que les unités de soins intensifs hautement performantes ont exploités pour atteindre des taux exceptionnels d’ID+AIG.

**Conception:**

Nous avons mené une étude qualitative descriptive afin de guider les efforts d’amélioration des taux d’ID+AIG dans le cadre d’une initiative de déviation positive.

**Cadre:**

Trois unités de soins intensifs hautement performantes ont été désignées comme sites d’étude.

**Participants:**

Les cliniciens exerçant dans les unités de soins intensifs identifiées.

**Méthodologie:**

À chaque site, nous avons interrogé des membres de l’équipe clinique sur leurs perceptions et leurs expériences d’ID+AIG. L’analyze des données a suivi une approche d’analyze thématique.

**Résultats:**

Nous présentons trois thèmes décrivant la manière dont les hôpitaux les plus performants parviennent à mettre en place de solides pratiques d’ID+AIG. Premièrement, toutes ces unités démontrent un degré élevé d’intégration entre les concepts de soins de fin de vie de haute qualité et le don d’organes. Les membres de l’équipe sont informés sur une base régulière des greffes réussies provenant de leur unité et toutes les occasions d’ID+AIG manquées font l’objet d’un suivi et de discussion. Deuxièmement, les participants ont décrit une approche d’équipe, menée par un solide leadership médical, où tous les membres assument leur rôle en s’assurant qu’aucun donneur potentiel ne soit manqué. Enfin, nous avons constaté que les unités hautement performantes adoptent des stratégies visant à soutenir et à simplifier l’ID+AIG, comme la simplification collective des critères d’aiguillage, le développement de solides relations de travail avec les coordonnateurs de dons provinciaux et la création de voies de communication informelles entre les cliniciens et les coordonnateurs de dons.

**Limites:**

L’absence de données comparables sur les taux d’aiguillage des donneurs d’organes potentiels au Canada a limité notre capacité à identifier les hôpitaux les plus performants à partir des données. Nous avons plutôt communiqué directement avec les OPDO pour identifier les unités les plus performantes répondant à nos critères. Aussi, notre échantillon était limité à trois hôpitaux de trois provinces différentes, qui procèdent tous au prélèvement et à la transplantation d’organes sur place.

**Conclusion:**

Les unités de soins intensifs peuvent adopter des stratégies et mettre en œuvre des interventions pour soutenir les efforts d’amélioration des taux d’ID+AIG. Notre étude en fournit des exemples. Nous mettons également en évidence les aspects qui nécessitent une attention particulière lorsqu’on s’engage dans cette voie; notamment s’assurer que tous les membres de l’équipe sont au courant des changements apportés aux plans de soins et que les médecins participent systématiquement aux discussions sur le don d’organes. Du leadership médical local est essentiel pour soutenir ces changements.

## Introduction

Deceased organ donation accounts for more than 80% of all solid organ transplants in Canada.^1^ Despite improvements in organ donation rates over the past decade, there continues to be a gap between those waiting for an organ and the number of organs available for transplantation across Canada.^2^ Critical to increasing the number of organs available for donation is ensuring that clinical team members identify all potential organ donors before arrest of circulation, and subsequently make a timely referral to the provincial organ donation organization (ODO).^3-6^ Predetermined identification and referral (ID&R) criteria facilitate this process^7^; however, the organ donor ID&R remains a complex, multistep process occurring in busy clinical settings with negotiation and cooperation required of the clinical team. These factors contribute to the potential for missed ID&R.^8-10^

Over the last 2 decades, there have been efforts to formalize and streamline the ID&R process at national and provincial levels, including the introduction of mandatory referral legislation,^11^ establishment of a national definition for potential organ donor,^7^ and the development of standardized data collection definitions and clinical triggers for the ID&R of potential donors.^11^ As of 2018, all provinces with the exception of Saskatchewan and Newfoundland have implemented or are implementing some sort of mandatory referral criteria. This can be a powerful instrument to encourage donation but is not sufficient. Broad variation remains within jurisdictions that have adopted such policies, with some hospitals demonstrating exceptional performance, despite experiencing similar constraints associated with existing structures, policies, and practices.^9,12^ This variability in performance highlights the importance of examining local characteristics and practices on ID&R outcomes and the need to focus improvement initiatives locally.

Understanding how some hospitals achieve exceptional performance can provide new insights into strategies and interventions for improving local ID&R rates.^3,13^ A positive deviance approach allows for the identification and study of individuals or organizations that demonstrate exceptional performance on an outcome of interest.^13,14^ This approach leverages naturally occurring variation to identify potential avenues for improvement.^15^ As part of a positive deviance approach,^14^ we conducted a qualitative study to identify the enablers and specific strategies that three identified high-performing sites, in provinces with mandatory referral legislation, leveraged to achieve their exceptional performance to inform ID&R improvement efforts in hospitals across Canada.

## Methodology

Baxter et al^15^ outlines 4 steps in their positive deviance approach: (1) identify high-performing cases using performance data, (2) conduct an in-depth study of the identified cases, using qualitative methods to generate hypotheses about strategies they apply to maintain high performance, (3) test the hypotheses using quantitative methods, with a larger generalizable sample, and (4) disseminate the strategies to the wider community. This article outlines the first 2 steps of this positive deviance approach. First, we identified positively deviant sites and second, we conducted a qualitative descriptive interview study of the 3 identified sites^16^. For step 3 and 4 of the positive deviance approach, the findings of this study will, in part, inform the creation of a toolkit to support ID&R improvement efforts. The toolkit will be implemented and tested through a national multisite improvement initiative, in partnership with Canadian Blood Services. Teams from participating organizations will use the toolkit to implement local changes, supported by project coaches to adapt recommendations to their local contexts, and measure the impact of these changes on their ID&R rates. By including multiple sites from across Canada, we will ensure that the toolkit will be tested in a range of jurisdictions and settings with varying baseline ID&R rates.

The University of Toronto Research Ethics Board granted ethical approval (protocol number 41693).

### Step 1: Identifying Positively Deviant Sites

We aimed to identify 3 high-performing critical care units across 3 provinces with mandatory referral legislation as study sites. We initially reached out to provincial ODOs for critical care unit ID&R rates; however, comparable data were not available across provinces, due to different reporting metrics. Instead, we contacted ODO Medical Directors in 3 high-performing provinces (Ontario, British Columbia, and Manitoba) to identify high-performing critical care units with referral rates of at least 85% that aligned with our sampling strategy, to ensure a range of characteristics across our sample. Characteristics included: (1) size of province and associated ODO, (2) whether the hospital had an onsite organ donor coordinator, (3) whether the hospital conducts organ retrieval procedures in-house, and (4) hospital size and type (academic vs community). Table 1 outlines the 3 participating critical care units’ characteristics.

**Table 1.** Site Characteristics and Interview Participants.

Site description	Relationship with ODO	Services available	Interview participants
Site 1 Hospital type: Large academic tertiary hospital Province: Manitoba	Provincial ODO office onsite and one dedicated coordinator	Recovery and transplantation	1 Intensive Care Unit (ICU) Medical Director 1 Donor Coordinator 1 ICU Physician 2 ICU Patient Care Managers
Site 2 Hospital type: Large academic tertiary hospital Province: Ontario	Two dedicated ODO coordinators onsite	Recovery and transplantation	2 Critical Care Medical Directors 2 Donor Coordinators 1 Clinical Educator 1 ICU Patient Care Manager
Site 3 Hospital type: Large community hospital Province: British Columbia	No onsite coordinator, one assigned coordinator	Recovery and transplantation	1 ICU Medical Director 1 ICU Physician 1 Donor Coordinator 1 Nurse Educator 1 ICU Patient Care Manager

### Step 2: Qualitative Descriptive Interview Study

#### Study participant sampling and data collection

At each of the participating sites, we used purposive and snowball sampling approaches to identify potential participants from diverse professional, clinical, and administrative groups.^17^ The principal author, LR, a PhD trained qualitative health researcher, who had no prior relationship with the participants, conducted the interviews by telephone or videoconference based on participant preference. We began by inviting the hospital’s dedicated organ donor coordinator (henceforth referred to as coordinators) and a physician with an organ donation leadership role through email and conducted 1-on-1 interviews virtually. During these interviews, we asked them to identify 4 to 6 other individuals who could provide insight about the hospital’s organ donation process and context (a total of 22 potential participants). Interviews were adapted based on clinical role, but broadly addressed perceptions of ID&R processes and organizational context and factors impacting ID&R performance. The interview guide is available as Appendix 1. Following consent, we audio-recorded and transcribed interviews using a professional third-party transcription service.

#### Data analysis

Data analysis followed a thematic analysis approach.^18^ Leahora Rotteau read all transcripts from the first site to become familiar with the data. Based on these data, a series of initial codes were generated and discussed with the authorship team. A coding framework was developed collectively and applied to all site 1 transcripts by LR. The coding framework included codes related to professional roles and ID&R, organizational context, ID&R processes and structures, leadership, messaging, and commitment. Discussions related to the coded data with the authorship team contributed to iterations to the coding framework. Leahora Rotteau subsequently applied the updated coding framework to the transcripts from the other 2 sites. Coded data from the 3 sites were compared to identify common or divergent strategies and interventions to inform the identification of themes. QSR International Nvivo 11 software was used for data management. The authorship team met regularly to discuss analysis and interpretation of the data. Three themes were identified and named that outline how identified units maintain and support strong ID&R performance.

## Findings

We conducted 16 interviews from March to December 2022. Interviews ranged from 15 to 60 minutes (average 38 minutes). Participants included medical directors, coordinators, physicians, patient care managers, and clinical and nursing educators. See Table 1 for the list of participants and their roles from each site.

### Theme 1: Commitment to Providing End-of-Life Care That Encompasses Opportunities for Organ Donation

Across all three sites, participants shared that their units demonstrated a shared commitment to providing quality end-of-life care. Ensuring opportunities for patients and families to donate was an integral component of, rather than in tension with, high-quality end-of-life care. They described ID&R as engrained in clinical practices. One coordinator from site 3 explained, ID&R is “part of their job” not an add-on.

Ultimately, the culture of it is that [organ donation is] part of patient care. I think it’s been engrained in our care. (14, Site 3, Medical Director)

When you’re approaching end-of-life, you also think about [name of ODO]. (6, Site 2, Clinical Educator)

Participants described formal ways their organizations incorporate ID&R into end-of-life care such as hospital policies around mandatory referral and end-of-life checklists that include organ referral. Though these formal structures and practices add legitimacy to the importance of ID&R, many participants shared other ways this commitment was supported. As one participant noted, “there is a policy, but I don’t think that staff are even really aware that it exists.” Education emphasizing the importance of organ donation at end-of-life was highlighted, including general nursing or critical care clinician education as well as real-time education following a missed referral to continuously reinforce ID&R practices at end-of-life.

We are looking to reframe it in a way that every [person] who dies should be offered the opportunity to donate. So, regardless of what an individual thinks that person’s ability to donate is, that is not up to them. We want to focus on that gift of the possibility of donation. That has worked well with nurses, in nurse educating focusing on that. (1, Site 1, Medical Director)

Whenever I come to [name of medical director] with any misses he follows up with physicians at that site so it’s become the culture of all the physicians there to call everything in. (12, Site 3, Coordinator)

At the three sites, clinical teams were consistently notified of successful transplants, stemming from a completed ID&R from their unit. This was seen by participants as a strategy to consistently reinforce that ID&R during end-of life can have positive outcomes and should be considered a component of high-quality end-of-life care. The notification letters generally outlined some clinical and demographic characteristics of the donor to bring the case to the team’s memory, and the number and types of transplants that occurred as a result of the unit’s referral. These letters also allow the clinicians on the unit to see “the good that can come out of the tragedy.”

I think the nurses really appreciate [the letter]. It gives you the conclusion of what exactly happened and who it helped and makes it feel positive. (6, Site 2, Clinical Educator)

Finally, missed referrals were tracked and reviewed at all 3 sites, reinforcing the importance of ID&R at end-of-life. Data from the ODO are shared directly with the clinical team. Different strategies were employed to review all deaths to identify missed referrals and investigate potential root causes, often led by or with support from the ODO. Nurse educators and physician leaders would ensure that there was discussion regarding strategies to improve ID&R.

Usually, the manager will speak to the staff that was maybe on that day or was involved and then if there is quite a few happening then we will try to book lunch-and-learns or a mini-education session. (2, Site 1, Coordinator)

The last couple months have been 100%. So, sometimes it’s just sending [the report] to the staff to be like “great job we’ve made all the appropriate referrals the last month.” (13, Site 3, Clinical Educator)

### Theme 2: Team Approach to ID&R Driven by Medical Leadership

All 3 sites described a team approach to ID&R, where all team members play a role in ensuring that no potential donor is missed. Specific to the identification of potential donors, participants described team bedside discussions about patient status and care plans where all team members were encouraged and had a shared responsibility to raise donation or ask if it was an appropriate time to make the referral. Bedside nurses were described as the group most likely to make the referral. However, it was noted that it is considered a shared responsibility, and others may make the referral to ensure that it is completed in a timely manner, such as the charge nurse or nursing educator. Beyond the nursing group, teams also noted that social workers or respiratory therapist can support identification by being aware of planned family meetings or changes in ventilation status. A unit manager^7^ from site 1 explained that “you have to get the whole team to come together first, to understand the plan for the patient” to support ID&R.

A physician leader^10^ from site 2 outlined the team-based approach in their critical care area as “having redundancy and having a shared responsibility.” The physician further explains:

[It’s] helpful to reinforce to the nurses that they are actually independently able to say this is heading this way and maybe we should call [the ODO]. They don’t have to wait for physicians to say that right away. (8, Site 2, Physician Leader)

Strong physician leadership related to organ donation fuels the clinical team’s collective commitment to organ donation and a team-based approach. First, across all sites, it was clear that the critical care medical leaders held a strong personal commitment to organ donation and encouraged the rest of the physician team to support ID&R. Physician leaders maintained strong relationships with the ODO and coordinators, through formal and informal communication. The coordinators at each site shared different ways they interacted with physician leaders and the importance of maintaining those relationships.

When asked about their organization’s commitment to organ donation, a Site 3 nurse educator explained that she does not “think there’s any huge organisation aspect that makes it a successful program. I think the fact that it’s mainly physician-driven, driving the importance of it” underscoring the importance of local leadership in supporting effective ID&R. Similarly, coordinators from Sites 1 and 3 echo the physician support for organ donation at their sites.

That portion really comes down to [name of medical director]. [Name of medical director] is an amazing advocate for organ donation and it really comes from the top. (12, Site 3, Coordinator)

There is major support for organ donation within critical care medical leadership. (3, Site 1, Coordinator)

Although critical care physicians are generally not the ones to complete the referral in practice, their actions had an impact on how the team functioned in relation to ID&R. Communicating with the team about the care plan and being open to conversations about donation in team rounds and other communications enabled the clinical team to identify potential organ donor candidates in a timely manner. The following quote from a physician leader at Site 2 illustrates the physician role:

If the physician [is] independently, without telling anyone, going, and having a talk with the family and coming out and saying, time to go to comfort care and we’d like to do it in an hour, that’s counterproductive. So, the physician’s role is equivalent to the huddle concept, to bring it even further upstream to say to the team, I think this is where we’re going. (8, Site 2, Physician Leader)

### Theme 3: Adoption of Strategies to Simplify and Support ID&R

Local efforts to make the ID&R process as easy as possible through a variety of mechanisms was seen as supporting successful ID&R. In this section, we describe two broad ways high-performing hospitals streamlined ID&R processes; simplifying the trigger for identification of potential donors and building working relationships with the coordinators.

First, participants discussed the benefits of simplifying the process associated with identifying potential donors. Each province has an established set of identification triggers and most clinicians we interviewed were able to recite them. However, when discussing how ID&R actually happens in practice, participants described simplifying those triggers. Most often, identification occurred when it was decided that a ventilated patient would move to comfort or end-of-life care. One physician leader explained that as a unit they have streamlined the ID&R process so that “any intubated, critically injured patient that’s approaching end-of-life, as part of a nursing checklist is automatically referred.” (1, Site 1). Adopting a more simplified trigger for identification was seen as enabling greater referral rates among the nursing group, due to an increased confidence in ability to identify potential donors.

In addition, this simplified trigger reduced the cognitive load associated with ID&R. For example, physicians were not required to consider the more nuanced inclusion and exclusion criteria for organ donation. Reinforcing the message that clinicians are simply responsible for making a referral once a ventilated patient transitions toward end-of-life care and leaving the assessment of eligibility to the coordinator and/or donor physicians was helpful in ensuring that all potential donors are identified and referred.

We used to decide whether they were appropriate, now it’s just easier to have other people make that decision . . . it eventually reduces the cognitive load in a day to say, we’re converting to comfort care and let them sort out that piece of the puzzle. (8, Site 2, Physician Leader)

I’d like to think that it’s maybe more on nursing that are actually taking the initiative and making the referral, because we know there’s no harm in it. You can do it without talking to the family. (12, Site 3, Coordinator)

Second, participants described strong working relationships with the coordinators. Coordinators described the importance of acting as an ally and someone who can make the work easier for clinicians as being a critical part of their role in supporting the clinical teams to consistently identify and refer potential donors. A coordinator for Site 3 explained:

I try to make it as easy as possible for physicians and for everybody on site. I want to support my hospitals as much as I can so that they’re not kind of like, ah, we’ve got to call this in and it’s a big hassle . . . when I go on site, they know that anybody can approach me and ask me any questions about donation. I’m pretty transparent about anything and happy to answer anything. I keep a very positive, friendly relationship with [the clinical team]. (12, Site 3, Coordinator)

Coordinators also described engaging in active monitoring for potential donors, such as daily conversations with the charge nurse or the social worker to monitor for upcoming family meetings where advanced care planning or end-of-life care might be discussed.

I would swing by the charge nurse, and I would ask them who’s have a goals of care discussion and then if she said bed 19 then I would go to the nurse at bed 19 and chat with them as well. (4, Site 2, Coordinator)

Finally, coordinators shared the benefits of less formal avenues for direct communication with clinicians to ease the burden of monitoring potential donors such as sharing phone numbers and texting questions.

Making relationships with social workers is important, saying, you can text me if you have a family meeting coming up, that’s perfect. I’m like, text me, I don’t mind, that’s super easy, it’s less time for you. Then it will help with physicians, during rounds they’re texting, saying things. (3, Site 2, Coordinator)

## Discussion

Through this qualitative study of 3 critical care programs across Canada, we identified 3 themes that outline ways critical care units can maintain and support strong ID&R performance. We describe the importance of making organ donation opportunities an integral element of end-of-life care, a team-based approach driven by local physician leaders, and unit-based efforts to simplify ID&R processes supported heavily by coordinators. These broad themes can inform concrete interventions or practices that critical care units can adopt to make improvements related to ID&R at the local level.^19^ Table 2 outlines a series of interventions and strategies informed by our findings that can be adopted by critical care units. As part of our ongoing positive deviance approach to support improvement of ID&R rates in critical care units in Canada, these identified interventions and strategies informed the development of a toolkit to support ID&R quality improvement. We will implement and test the toolkit through a multisite improvement initiative in critical care units across the country.

**Table 2.** List of Interventions to Improve Local ID&R Performance.

Theme 1: Commitment to providing high quality end-of-life care that encompasses opportunities for organ donation	
**Aim:** Ensuring all families are offered the opportunity of organ donation as part of high-quality end-of-life care	
Intervention	Description
Education inclusive of family perspectives	Reinforces organ donation as part of high-quality end-of-life care and acknowledges social norms related to ID&R
Script for the clinical team to support conversations with families	Supports clinicians informing the patient’s family that an organ donation coordinator will be meeting with them to discuss the opportunity of organ donation
Letter to the clinical team	Highlight to the care team who cared for the patient, how many people benefited from organ donation to reinforce the importance of ID&R
Review patient deaths	Data from ODO are shared directly with clinical teams and to identify improvement and education opportunities
Integrate ID&R with end-of-life clinical workflow	Pair end-of-life care and withdrawal of life sustaining treatment with organ donation
Theme 2: Team approach to ID&R driven by medical leadership	
**Aim:** Empowering and supporting all team members to play an active role in ID&R	
Intervention	Description
Relationships and communication between team members	Strong relationships between physician leadership, ODO and coordinators
Bedside discussions about patient status and care plans	Ensure all team members are included in, or notified about, patient status and changes in care plans
Simulation training	Prepare team members on how best to communicate with families that an organ donor coordinator will be meeting them to discuss the opportunity of organ donation
Theme 3: Adoption of strategies to simplify and support ID&R	
**Aim:** To make ID&R easier for the clinical team	
Intervention	Description
Separate clinical team from the organ donation conversation	Offload some of the ID&R work from clinical personnel
Active monitoring for potential donors	Encourage team members and coordinators to actively monitor for potential donors (e.g., tracking upcoming family meetings)
Team agreement on a simplified trigger	Reduce the mental effort involved in ID&R by adopting ventilated and end-of-life family meeting planned or discussed as a simplified trigger

The benefits of providing families the opportunity to donate organs as an integral part end-of-life care have been described by others.^20,21^ Our findings are consistent with other research demonstrating that offering the opportunity of organ donation is seen as beneficial to both families and critical care staff following the death of a patient and helps to solidify its value as part of high-quality end-of-life care. Teams were consistently reminded that ID&R provides the gift of the *possibility* of donation. We found that local leadership, especially medical leadership, is critical to reinforcing this philosophy. Our study provides examples of strategies to bolster this connection, such as letters to clinicians describing positive outcomes related to their efforts to ID&R, dedicated education, sharing timely ID&R data, and consistent follow-up when there is a missed referral.

Although offering the opportunity to donate is generally well accepted and promoted as part of end-of-life care, there are ethical and practical challenges that should be considered when developing local strategies.^22,23^ Experiences of the competing obligation to care for critically ill patients while maintaining best practices for organ donation at end-of-life, as well as perceptions of the impact of ID&R on clinical team members’ relationships with families, can add complexity to ID&R processes.^24^ The suggestion to streamline the ID&R process and refer ventilated patients as soon as they begin transitioning to end-of-life care can address this concern, as the coordinator can lead conversations regarding donation leaving the clinical team to continue providing patient care.

Although our findings suggest team-based approaches to organ donation ID&R are effective, prior research suggests many critical care settings may not support such practices. A team-based approach necessitates that members of the clinical team are aware of changes in care plans. This may not always be the case, with research demonstrating that in many settings nurses feel excluded from important care discussions.^25^ Efforts to improve communication between team members and the adoption of a collaborative end-of-life care framework^26^ may create more opportunities for discussions regarding ID&R which includes all team members. We found that physician leaders can play an important role in establishing a unit culture that is supportive of a team-based approach. However, research has shown variations in how physicians view organ donation and the process of ID&R, including the practice of mandatory referral.^27,28^ Our findings suggest supporting physicians’ roles in ensuring ID&R of potential donors through education or peer or leadership modeling, is an important strategy for critical care departments.

It has been suggested that clear role responsibilities and a standardized process for ID&R can facilitate consistency of this practice.^21^ However, our findings suggest that one of the clear benefits of a well functioning team-based approach to ID&R is the redundancy of encouraging multiple clinical team members to embrace the role of ensuring that no potential donors are missed. Simplifying the trigger for ID&R of potential donors and building a collective understanding of that trigger among the whole clinical team supports the team-based approach and may enable balancing the need for both standardization and redundancy to enable ID&R.

Last, our study underscores the role of dedicated coordinators in improving consistent ID&R, echoing other literature related to the organ donation process.^29,30^ The coordinators in our study emphasized that a central component of their role was making organ donation easier for the clinical teams, including ID&R. This was accomplished by developing accessible formal and informal avenues for communication leading up to the referral of a potential donor and active monitoring of potential donors.^33^ Not every critical care unit in Canada will have the same level of support from dedicated coordinators, but we suggest that many functions of this role could be replicated or adapted by other clinical team members, such as social workers, respiratory therapists, or nurse managers.

### Limitations

Our study has limitations. First, the lack of consistent data for potential organ donor referral rates across Canada impacted our ability to identify high-performing hospitals based on comparable data. Instead, we contacted ODOs directly to identify high-performing units that met our criteria. Second, our study sample was limited to 3 hospital sites from 3 different provinces. The 3 hospitals perform organ retrieval and transplant on site. However, we did seek variability on other factors related to ID&R, such as type and size of hospital (academic or community) and the presence of on-site coordinators. Finally, we did not interview participants from emergency departments, pediatrics or other clinical settings that may treat potential donors. Although some of the findings from this study may be transferable, further context-specific research is needed to ensure alignment of improvement strategies.

## Conclusion

This qualitative study of 3 high-performing critical care units illustrates ways these units maintained their high performance. Units consistently maintained a commitment to providing high-quality end-of-life care that encompasses opportunities for organ donation, embraced a team approach to ID&R driven by medical leadership and adopted strategies to simplify and support ID&R. Strategies included collectively simplifying triggers for referral, creating strong working relationships with provincial donor coordinators, and creating less formal avenues for communication between clinicians and donor coordinators to ease the burden of monitoring potential donors. Consistent with a positive deviance approach, these strategies and interventions will inform the development of a toolkit outlining a range of strategies that critical care units can adopt to improve ID&R rates. We have taken care to include strategies that can be adopted across different organizational context and resource limitations and have provided tools to determine best strategies to implement based on various contextual factors. The toolkit will be tested through a multisite improvement initiative and subsequently disseminated widely.

## Supplemental Material

sj-docx-1-cjk-10.1177_20543581241276362 – Supplemental material for Characteristics and Practices of High-Performing Centers in Organ Donor Identification and Referral: A Qualitative Study Supplemental material, sj-docx-1-cjk-10.1177_20543581241276362 for Characteristics and Practices of High-Performing Centers in Organ Donor Identification and Referral: A Qualitative Study by Leahora Rotteau, Samuel Vaillancourt, Mercedes Magaz, Lisha Lo, Brian M. Wong, Jehan Lalani, Sam D. Shemie and Samara Zavalkoff in Canadian Journal of Kidney Health and Disease

sj-docx-2-cjk-10.1177_20543581241276362 – Supplemental material for Characteristics and Practices of High-Performing Centers in Organ Donor Identification and Referral: A Qualitative Study Supplemental material, sj-docx-2-cjk-10.1177_20543581241276362 for Characteristics and Practices of High-Performing Centers in Organ Donor Identification and Referral: A Qualitative Study by Leahora Rotteau, Samuel Vaillancourt, Mercedes Magaz, Lisha Lo, Brian M. Wong, Jehan Lalani, Sam D. Shemie and Samara Zavalkoff in Canadian Journal of Kidney Health and Disease
