# Supplementary material for: Characteristics and Practices of High-Performing Centers in Organ Donor Identification and Referral: A Qualitative Study
Source: Can J Kidney Health Dis. 2024 Sep 21;11:20543581241276362. doi: 10.1177/20543581241276362 (PMC11418359; doi:10.1177/20543581241276362)
Supplement: sj-docx-2-cjk-10.1177_20543581241276362 – Supplemental material for Characteristics and Practices of High-Performing Centers in Organ Donor Identification and Referral: A Qualitative Study [file sj-docx-2-cjk-10.1177_20543581241276362.docx]

**MDO Interview Guide**

**Introduction**

1. Can you describe your role with organ donation at [name of hospital]?

**Organ donor identification and referral process**

1. For this interview we are specifically interested in the identification and referral of organs for donation. From your perspective, can you walk me through that process?
   1. Is this typically what happens? Are there parts of the process that don’t always go as planned?
   2. Can you think of an organ donation referral that followed the expected process? What about an example of a donation referral that deviated from the process?
   3. Do you think there is a clear understanding of the process among clinicians?
   4. Do you think there is a clear understanding of who is eligible to be an organ donor?
2. Who is involved with the identification and referral process?
   1. Respiratory therapists, RNS, MDs, charge nurse, unit manager
   2. Are there any formal or informal clinical champions?
   3. Is there someone who has final authority/makes the final decision about referral?

**Organizational Factors**

1. Can you describe the relationship between your hospital and the provincial organ donation organization?
   1. Frequency and routes of interaction? Support?
2. How would you describe your organization’s commitment to organ donation? How would you describe your unit/department’s commitment to organ donation?
   1. How is this commitment demonstrated or not?

**Organ donation identification and referral performance enablers**

1. Your institution was flagged as a ‘high performer’ when it comes to identifying and referring potential organ donors. How do you think your institution achieved such high identification and referral rates?
2. What are the strategies at [name of hospital] that facilitates organ identification and referral?
   1. Forcing function, Automation, Standardization, Checklists and reminders, Rules and policies, Education and training?
3. What do you think are the biggest or ongoing challenges to organ identification and referral?
4. Are there any current or plan efforts to improve organ donor identification and referral rates at [name of hospital]?
5. Let’s say your institution is committed to never missing a referral. What do you think is the most effective way to achieve that?
6. Does [name of hospital] collect and share data related to tracking organ donation?
   1. How is the data used?
   2. Do you think that the data is accurate?
   3. Do you get feedback on your personal/department/hospital cases?

**Wrap-up**

1. Is there anything else about your experience with organ donor identification and referral that we have not yet discussed that you think would be helpful for us to know more about?
